# Diagnostic Ability of Quantitative Parameters of Whole-Body Bone SPECT/CT Using a Full-Ring 360° Cadmium-Zinc-Telluride Camera for Detecting Bone Metastasis in Patients with Prostate Cancer

**DOI:** 10.3390/diagnostics14232714

**Published:** 2024-12-02

**Authors:** Ik Dong Yoo, Sun-pyo Hong, Sang Mi Lee, Hee Jo Yang, Ki Hong Kim, Si Hyun Kim, Jeong Won Lee

**Affiliations:** 1 Department of Nuclear Medicine, Soonchunhyang University Cheonan Hospital, 31 Suncheonhyang 6-gil, Dongnam-gu, Cheonan 31151, Republic of Korea; 2 Department of Urology, Soonchunhyang University Cheonan Hospital, 31 Suncheonhyang 6-gil, Dongnam-gu, Cheonan 31151, Republic of Korea

**Keywords:** bone metastasis, bone scintigraphy, prostate cancer, quantification, single-photon emission computed tomography

## Abstract

Background/Objectives: This study aimed to assess the diagnostic capability of quantitative parameters from whole-body bone single-photon emission computed tomography/computed tomography (SPECT/CT) in detecting bone metastases in prostate cancer patients; Methods: We retrospectively analyzed 82 prostate cancer patients who underwent staging bone scintigraphy with a full-ring 360° Cadmium-Zinc-Telluride (CZT) SPECT/CT system. From the SPECT/CT images, we measured the maximum (SUVmax) and mean (SUVmean) standardized uptake values at six normal bone sites (skull, humerus, thoracic spine, lumbar spine, iliac bone, and femur), and the SUVmax for both metastatic and benign bone lesions. Ratios of lesion SUVmax-to-maximum and mean uptake values at the skull, humerus, and femur were computed for each lesion; Results: SUVmax and SUVmean at the skull and femur exhibited significantly lower variance compared to those at the thoracic spine, lumbar spine, and iliac bone, and revealed no significant differences between patients with and without bone metastasis. In receiver operating characteristic curve analysis for detecting bone metastasis among 482 metastatic lesions, 132 benign bone lesions, and 477 normal bone sites, the lesion-to-femur mean uptake ratio demonstrated the highest area under the curve value (0.955) among SPECT/CT parameters. Using a cut-off value of 5.38, the lesion-to-femur mean uptake ratio achieved a sensitivity of 94.8% and a specificity of 86.5%; Conclusions: The bone lesion-to-femur mean uptake ratio was the most effective quantitative bone SPECT/CT parameter for detecting bone metastasis in prostate cancer patients. Quantitative analysis of bone SPECT/CT images could thus play a crucial role in diagnosing bone metastasis.

## 1. Introduction

Prostate cancer ranks as the second most common cancer and the fifth leading cause of cancer-related death worldwide in men [1]. Bone is the most common site of distant metastasis for prostate cancer [2]. The incidence of bone metastasis among prostate cancer patients has been reported to range between 4.4 and 11.9% at the time of diagnosis, highlighting the importance of imaging examinations for bone metastasis in the staging work-up of prostate cancer [2,3,4]. Typically, bone metastasis from prostate cancer induces an osteoblastic reaction, with over 90% of cases developing osteoblastic lesions [5,6]. Since ^99m^Tc-labelled diphosphonate compounds accumulate in the skeletal system in proportion to bone vascularity and osteoblastic activity, planar bone scintigraphy using ^99m^Tc-labelled diphosphonate compounds has been the most commonly employed imaging technique for evaluating bone metastasis in prostate cancer patients [4,5,7,8]. However, although planar bone scintigraphy is highly sensitive for detecting bone metastasis and permits assessment of the entire skeletal system with a single scan, it lacks specificity and a quantitative objective assessment method [5,8,9].

Bone single-photon emission computed tomography/computed tomography (SPECT/CT) is an imaging modality that produces three-dimensional images by combining planar bone scintigraphy with CT images. It enhances the anatomical localization of bone lesion and more definitively differentiates between bone metastasis and benign bone lesion; therefore, it demonstrates superior diagnostic performance in detecting bone metastasis with less equivocal results compared to planar bone scintigraphy [8,10]. Recently, a new SPECT/CT system equipped with full-ring 360° Cadmium-Zinc-Telluride (CZT) detectors has been introduced [11]. Compared to conventional dual-head SPECT/CT with sodium iodide crystal detectors, this new system utilizes CZT detectors arranged in a 360° ring configuration geometry, enabling very close positioning to the patient’s body [11,12]. This innovative geometry optimizes energy and contrast resolution and enhances count sensitivity, dramatically reducing the whole-body SPECT image acquisition time from 40–50 min to 22 min [11,12,13]. Furthermore, it enables a quantitative analytic method for estimating ^99m^Tc-labelled diphosphonate compound uptake in bone lesions by calculating the standardized uptake value (SUV) [12,14]. The quantitative SPECT/CT imaging parameters provide an objective measure for assessing bone lesions, which is invaluable for diagnosing bone metastasis [15]. However, to date, only a limited number of studies have assessed the diagnostic role of quantitative imaging parameters from CZT SPECT/CT images in characterizing bone lesions [12,14,15].

Therefore, the current study aimed to investigate the diagnostic ability of quantitative imaging parameters in whole-body bone SPECT/CT using a full-ring 360° CZT camera for detecting bone metastasis in patients with prostate cancer.

## 2. Materials and Methods

### 2.1. Patients

We retrospectively reviewed patients who were histopathologically diagnosed with prostate cancer and underwent bone scintigraphy for staging work-up using a full-ring 360° CZT SPECT/CT system at our medical center between April 2021 and March 2024. Among these patients, we included patients who had radiographic confirmation of bone SPECT/CT findings by additional diagnostic imaging examinations for staging work-up, such as magnetic resonance imaging, F-18 sodium fluoride bone positron emission tomography (PET)/CT, and F-18 fluorodeoxyglucose PET/CT, and/or follow-up imaging studies. Patients were excluded if they (1) had a prior history of another malignant disease, (2) had received any treatment before bone SPECT/CT, or (3) did not undergo additional staging or follow-up imaging studies; thus, confirming bone SPECT/CT findings was not possible. Based on the inclusion and exclusion criteria, 82 patients with prostate cancer were ultimately enrolled in the current study.

### 2.2. Bone SPECT/CT Acquisition Protocol

All bone SPECT/CT images for the enrolled patients were acquired using the VERITON hybrid SPECT/CT scanner (VERITON-CT, Spectrum Dynamics Medical, Caesarea, Israel), which consists of 12 CZT detector columns arranged at 360° and a 16-slice CT. The SPECT/CT images were obtained 3 to 4 h after administering approximately 740 MBq of ^99m^Tc-methylene diphosphonate (MDP). The low-dose CT scan was initially conducted at the 100 kV and 30 mA settings. Subsequently, whole-body SPECT images were acquired in 6 to 7 bed positions, depending on the patient’s height, with each bed position being scanned for 100 s. The SPECT images were reconstructed using the ordered subset expectation maximization method with four iterations and four subsets. Image correction was performed using both the point spread function method and a partial volume correction algorithm, incorporating anatomical information from the CT images.

### 2.3. SPECT/CT Image Analysis

**Figure 1:**
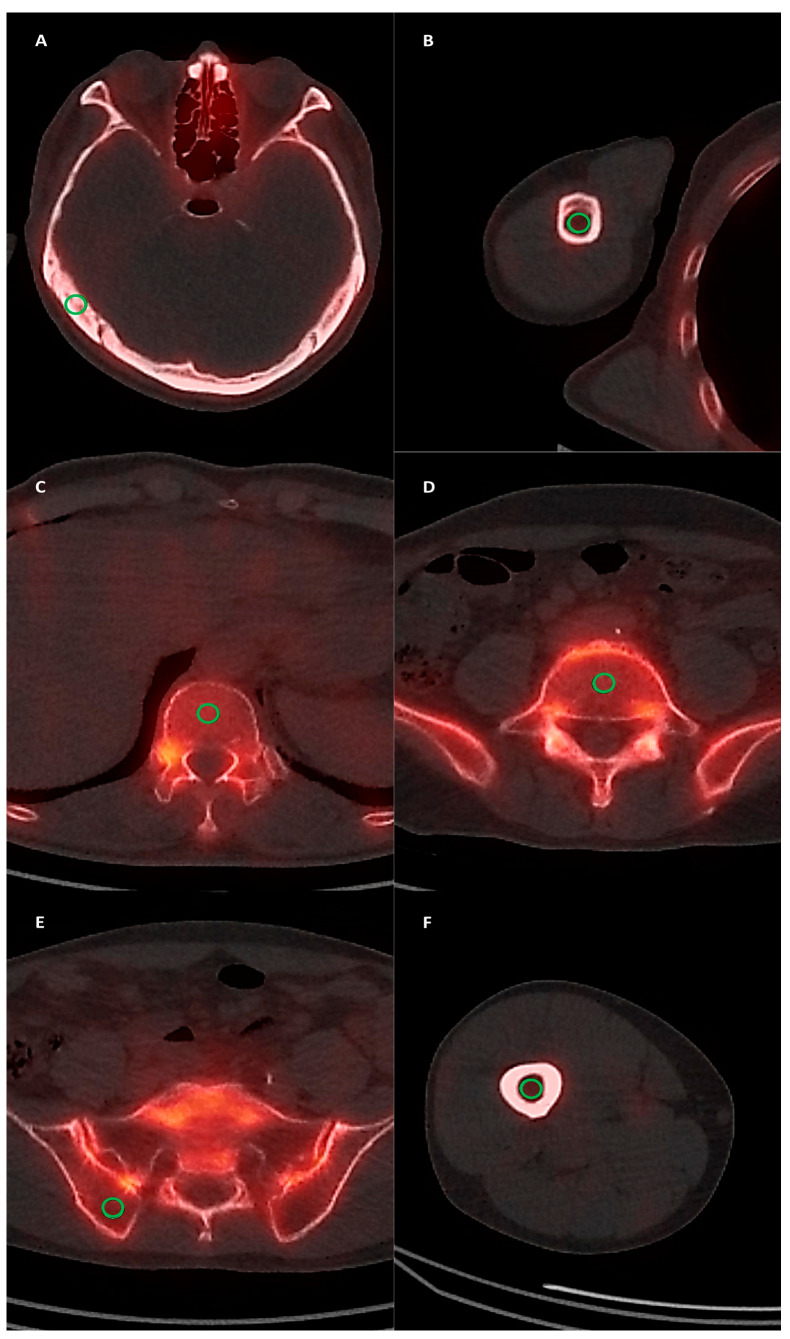
Examples of normal bone uptake measurements at the skull (**A**), humerus (**B**), thoracic spine (**C**), lumbar spine (**D**), iliac bone (**E**), and femur (**F**). A 1 cm-sized spherical VOI was manually drawn (green circle), and the SUVmax and SUVmean of the VOI were measured.

Bone SPECT/CT images were retrospectively assessed by three experienced nuclear medicine physicians through consensus using MIM software version 7.3.7 (MIM Software Inc., Cleveland, OH, USA). Initially, the degree of non-pathological normal bone uptake was measured at six normal bone sites (skull, humerus, thoracic spine, lumbar spine, iliac bone, and femur) in each patient. Areas exhibiting only mild diffuse uptake without focal uptake and anatomical abnormalities on CT images were selected for measuring normal bone uptake. A 1 cm-sized spherical volume of interest (VOI) was manually drawn over the temporal bone, the shaft of the humerus, vertebral bodies of the 12th thoracic and 5th lumbar spines, posterior iliac bone, and the shaft of the femur in SPECT/CT images (Figure 1), and the maximum standardized uptake value (SUVmax) and mean SUV (SUVmean) of the VOI were measured. In patients with bone metastasis, post-operative changes, compression fractures, or severe osteoarthritic changes in the 12th thoracic spine or 5th lumbar spine, the VOI was drawn at alternative thoracic or lumbar spine sites. Afterwards, abnormal bone lesions on SPECT/CT that showed radiotracer uptake exceeding normal bone uptake were identified and classified into metastatic and benign bone lesions based on additional staging and follow-up imaging studies. VOIs were manually outlined around the identified bone lesions, and the SUVmax was calculated for all metastatic and benign bone lesions. Finally, the SUVmax of each bone lesion was divided by the SUVmax and SUVmean of the skull, humerus, and femur, resulting in the calculation of the lesion-to-skull maximum uptake ratio, lesion-to-skull mean uptake ratio, lesion-to-humerus maximum uptake ratio, lesion-to-humerus mean uptake ratio, lesion-to-femur maximum uptake ratio, and lesion-to-femur mean uptake ratio, respectively.

### 2.4. Statistical Analysis

Clinical characteristics and uptake at normal bone sites were compared between patients with and without bone metastasis using the Mann–Whitney U test, chi-square test, and Fisher’s exact test. An F-test with Bonferroni correction was employed to compare the variance in uptake across six normal bone sites (skull, humerus, thoracic spine, lumbar spine, iliac bone, and femur). The Kruskal–Wallis test, followed by post hoc comparisons using Dunne’s test, assessed differences in seven SPECT/CT imaging parameters (SUVmax, lesion-to-skull maximum uptake ratio, lesion-to-skull mean uptake ratio, lesion-to-humerus maximum uptake ratio, lesion-to-humerus mean uptake ratio, lesion-to-femur maximum uptake ratio, and lesion-to-femur mean uptake ratio) among metastatic bone lesions, benign bone lesions, and normal bone sites. The diagnostic efficacy of these seven SPECT/CT imaging parameters in identifying metastatic bone lesions was evaluated based on the area under the receiver operating characteristic (ROC) curve (AUC) values. The sensitivity and specificity for each parameter were determined using optimal cut-off values derived from the Youden index. Statistical analyses were performed using MedCalc Statistical Software (version 23.0.2, MedCalc Software Ltd., Ostend, Belgium), and a *p*-value of <0.05 was deemed indicative of statistical significance.

## 3. Results

### 3.1. Patient Characteristics

The clinical characteristics of the 82 enrolled patients with prostate cancer are presented in Table 1. In the assessment of normal bone uptake, uptake in the skull, humerus, and femur was successfully measured in all 82 patients; however, due to the presence of disseminated bone metastases, uptake in the iliac bone, thoracic spine, and lumbar spine was measured only in 78 patients, 76 patients, and 77 patients, respectively. Consequently, SUVmax and SUVmean at normal bone sites were measured in a total of 477 sites. In all patients, 614 bone lesions were identified, consisting of 482 metastatic bone lesions (78.5%) and 132 benign bone lesions (21.5%). These 482 metastatic bone lesions were identified in 31 patients. The benign bone lesions included 82 degenerative changes (13.4%), such as osteoarthritis and facet joint arthropathy, 48 post-traumatic lesions (7.8%), and 2 benign bone tumors (0.3%). In comparisons of clinical characteristics between patients with and without bone metastases, the 31 patients with metastatic bone lesions exhibited significantly higher serum prostate-specific antigen levels and Gleason grade group and advanced tumor stage (*p* < 0.05; Table 1).

### 3.2. Normal Bone Uptake of SPECT/CT Images

**Figure 2:**
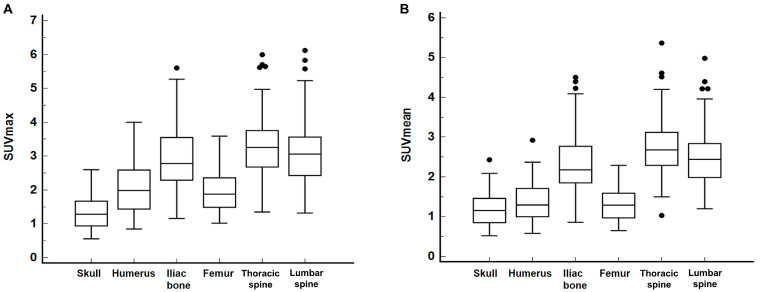
Distribution of SUVmax (**A**) and SUVmean (**B**) at six normal bone sites: skull, humerus, iliac bone, femur, thoracic spine, and lumbar spine (black dot: an outside value which is larger than the 75 percentile value plus 1.5 times the interquartile range).

The distributions of SUVmax and SUVmean at normal bone sites are displayed in Table 2 and Figure 2. In comparing the variance of SUVmax among normal bone sites using the F-test with Bonferroni correction, both the skull (1.32 ± 0.45) and femur (1.96 ± 0.58) exhibited significantly smaller variance than the thoracic spine (3.33 ± 0.94), lumbar spine (3.14 ± 0.97), and iliac bone (2.96 ± 0.95) (*p* < 0.05). The variance of SUVmax in the humerus (2.07 ± 0.70), however, did not significantly differ from that of other normal bone sites (*p* > 0.05). In terms of SUVmean, the skull (1.17 ± 0.41), humerus (1.36 ± 0.48), and femur (1.29 ± 0.40) also demonstrated significantly smaller variance compared to the thoracic spine (2.74 ± 0.76), lumbar spine (2.49 ± 0.75), and iliac bone (2.36 ± 0.78) (*p* < 0.05).

We further explored the SUVmax and SUVmean differences in normal bone sites between patients with and without bone metastasis (Table 2). For the skull, humerus, and femur, no significant differences in SUVmax or SUVmean were observed between the two patient groups (*p* > 0.05). In contrast, patients with bone metastasis demonstrated significantly higher SUVmax values in the thoracic spine, lumbar spine, and iliac bone, and elevated SUVmean values in the lumbar spine compared to those without bone metastasis (*p* < 0.05). SUVmean values for the thoracic spine and iliac bone also tended to be higher in patients with bone metastasis, reaching borderline statistical significance (*p* < 0.10).

### 3.3. Diagnostic Ability of SPECT/CT Imaging Parameters

Since the uptake in the skull, humerus, and femur exhibited narrower value distribution compared to other normal bone sites and was unaffected by the presence of bone metastasis, ratios between the SUVmax of bone lesions and the uptake (both SUVmax and SUVmean) in the skull, humerus, and femur were calculated to reduce inter-individual variations. Consequently, seven SPECT/CT imaging parameters (SUVmax, lesion-to-skull maximum uptake ratio, lesion-to-skull mean uptake ratio, lesion-to-humerus maximum uptake ratio, lesion-to-humerus mean uptake ratio, lesion-to-femur maximum uptake ratio, and lesion-to-femur mean uptake ratio) were incorporated into the analysis to evaluate diagnostic capabilities in detecting bone metastasis. The Kruskal–Wallis test demonstrated significant differences across all seven parameters among metastatic bone lesions, degenerative changes, post-traumatic lesions, and normal bone sites (*p* < 0.001; Table 3). In post hoc analysis, values in metastatic bone lesions were significantly higher than those in benign bone lesions and normal bone sites for six SPECT/CT parameters, except for the lesion-to-humerus mean uptake ratio (*p* < 0.05). For the lesion-to-humerus mean uptake ratio, values in metastatic bone lesions were significantly higher than those in degenerative changes and normal bone sites (*p* < 0.05), but there was no significant difference between metastatic bone lesions and post-traumatic lesions (*p* > 0.05).

**Figure 3:**
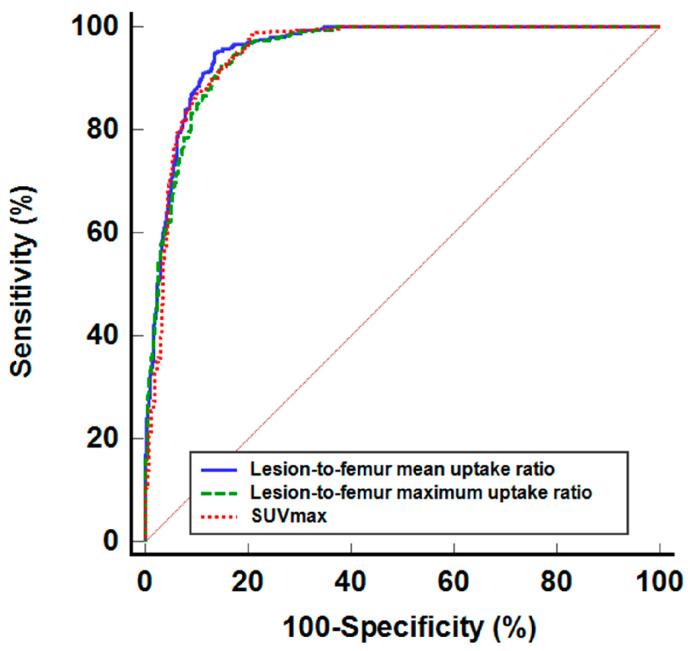
Receiver operating characteristic curves for the lesion-to-femur mean uptake ratio, lesion-to-femur maximum uptake ratio, and SUVmax in detecting metastatic bone lesion among metastatic and benign bone lesions and normal bone sites.

The detection abilities of seven SPECT/CT imaging parameters for bone metastasis among metastatic and benign bone lesions and normal bone sites are presented in Table 4. Of the seven parameters, the lesion-to-femur mean uptake ratio demonstrated the highest AUC value [0.955; 95% confidence interval (CI), 0.942–0.967], followed by the lesion-to-femur maximum uptake ratio (0.950; 95% CI, 0.935–0.962) and SUVmax (0.949; 95% CI, 0.935–0.962) (Figure 3). Utilizing an optimal cut-off value of 5.38, the lesion-to-femur mean uptake ratio achieved a sensitivity of 94.8% (95% CI, 92.4–96.6%) and a specificity of 86.5% (95% CI, 83.6–89.1%) for detecting bone metastasis. Comparisons of ROC curves indicated that the lesion-to-femur mean uptake ratio has a significantly higher AUC value than the lesion-to-skull maximum uptake ratio, lesion-to-skull mean uptake ratio, lesion-to-humerus maximum uptake ratio, and lesion-to-humerus mean uptake ratio (*p* < 0.05), while there were no significant differences between the lesion-to-femur mean uptake ratio, lesion-to-femur maximum uptake ratio, and SUVmax (*p* > 0.05).

**Figure 4:**
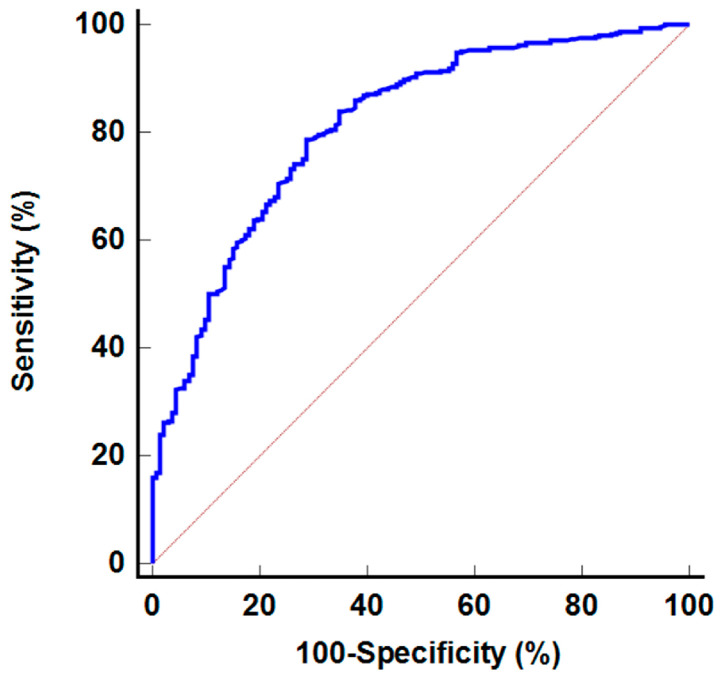
Receiver operating characteristic curve for the lesion-to-femur mean uptake ratio in detecting metastatic bone lesion among abnormal bone lesions.

We further evaluated the diagnostic abilities of seven SPECT/CT imaging parameters for detecting bone metastasis among abnormal bone lesions, which included only metastatic and benign bone lesions (Table 5). Again, the lesion-to-femur mean uptake ratio exhibited the highest AUC value (0.813; 95% CI, 0.780–0.843) among the seven SPECT/CT parameters (Figure 4). The sensitivity and specificity of this ratio were 80.1% (76.2–83.6%) and 67.4% (58.7–75.3%), respectively, using an optimal cut-off value of 7.89. In the ROC curve comparisons, the lesion-to-femur mean uptake ratio demonstrated a significantly higher AUC value than the other six SPECT/CT parameters (*p* < 0.05).

## 4. Discussion

In the present study, we explored the diagnostic value of quantitative parameters from staging bone SPECT/CT in detecting bone metastasis in 82 patients with prostate cancer. The results of our study demonstrated that the lesion-to-femur mean uptake ratio possessed the best diagnostic ability for detecting bone metastasis in prostate cancer patients, showing high sensitivity. These findings suggest that the uptake ratio by reference bone uptake, rather than SUVmax, could be more suitable for distinguishing between metastatic and benign bone lesions.

Previous studies have shown that ^99m^Tc-labelled diphosphonate compound uptake in normal bone is significantly influenced by various patient factors, such as age, sex, weight, height, and CT attenuation [16,17]. Consequently, both the SUVmax and SUVmean values of normal bone sites exhibit considerable variability among patients, which our study also demonstrated [5,15,16,17]. Therefore, we hypothesized that the uptake ratio between bone lesions and a reference normal bone site could minimize inter-individual variability, thus offering greater clinical relevance for diagnosing bone metastases than using SUVmax alone. Our findings suggest that the SUVmax and SUVmean of the skull and femur, along with the SUVmean of the humerus, exhibited significantly lower variance compared to the thoracic spine, lumbar spine, and iliac bone, indicating smaller discrepancies in radiotracer uptake among patients. Additionally, unlike the thoracic spine, lumbar spine, and iliac bone, the presence of bone metastasis did not affect the SUVmax and SUVmean values of the skull, humerus, and femur, corroborating results from a previous study of bone SPECT/CT in patients with prostate cancer [18]. Given these findings in the present study, the skull, humerus, and femur appeared to be viable reference sites for normal bone uptake in this context.

Previous meta-analyses encompassing 11 studies and 1611 patients with various malignant diseases have demonstrated that bone SPECT/CT has already been found to have high diagnostic accuracy for detecting bone metastasis, superior to planar bone scintigraphy with less equivocal results [8]. However, most of these 11 studies in the meta-analysis utilized conventional dual-head SPECT/CT with a limited field of view, and the results were primarily based on patient-based qualitative analysis [8]. Qualitative analysis, heavily reliant on the practitioner’s expertise and experience, lacks objective assessment [19]. Conversely, quantitative SPECT/CT analysis can enhance the certainty of image interpretation and improve the diagnostic accuracy for bone metastasis [19,20]. Nevertheless, despite the introduction of quantitative SPECT into the field of nuclear medicine several decades ago, the clinical utility of quantitative SPECT imaging parameters is still under investigation [7,21,22].

For patients with prostate cancer, only a few studies have assessed the diagnostic performance of quantitative imaging parameters from bone SPECT/CT for bone metastasis using a conventional dual-head SPECT/CT system equipped with sodium iodide crystal detectors [5,18,19]. One study, which involved 130 prostate cancer patients and used normal bone site uptake values as non-metastatic lesions, demonstrated that the sensitivity and specificity of SUVmax were 87.0% and 94.3%, respectively, with a cut-off SUVmax of 7.0 [18]. Another study that included 265 metastatic bone lesions and 24 osteoarthritic changes in 26 prostate cancer patients also demonstrated a high AUC value for SUVmax (0.947) with a sensitivity of 87% and specificity of 92% at a cut-off value of 19.5 [5]. By contrast, the other study assessing 48 metastatic and 40 benign bone lesions in 51 prostate cancer patients found a lower AUC value of only 0.687 for SUVmax in detecting bone metastasis [19]. The diagnostic potential of quantitative parameters from the CZT SPECT/CT system in patients with prostate cancer remains unreported, though a recent study evaluated SUVmax of metastatic bone lesions and normal bone sites using the dual-head CZT detector system [15]. Although it did not examine the diagnostic performance of SUVmax for bone metastasis directly, the study suggested an optimal SUVmax threshold of 8.0 for differentiating uptake between normal bone and bone metastasis, since only 1.0% of normal bone sites exceeded an SUVmax of 8.0 [15]. In the current study, metastatic bone lesions exhibited significantly higher SUVmax values compared to degenerative changes, post-traumatic lesions, and normal bone uptake. The SUVmax also demonstrated a high AUC value of 0.949 for distinguishing bone metastasis from benign bone lesions and normal bone sites, aligning with findings from the previous studies [5,18]. Furthermore, we calculated bone lesion-to-normal bone site uptake ratios and explored their potential to offer greater diagnostic value than SUVmax alone. Our findings demonstrated that the lesion-to-femur mean uptake ratio yielded the highest AUC value for detecting bone metastasis among the quantitative SPECT/CT parameters, surpassing that of SUVmax. The lesion-to-femur mean uptake ratio showed high sensitivity in differentiating bone metastasis from both benign bone lesions and normal bone uptake (94.8%) and solely from benign bone lesions (80.1%). These results suggest that relative thresholding, such as the lesion-to-femur mean uptake ratio, could be more effective in diagnosing bone metastasis than absolute thresholding methods like SUVmax. In a recent study, uptake volume and total uptake of lesions that exceeded the threshold SUVmax were found to be significantly associated with patient survival [15]. Consequently, our future research will focus on evaluating the prognostic significance of uptake volume and total lesion uptake delineated by the lesion-to-femur mean uptake ratio threshold to establish the clinical utility of the relative threshold method.

Several studies, including the current study, have reported absolute uptake values for normal bone sites, such as the thoracic and lumbar spines, although variations in SUVmax and SUVmean were observed across these studies [5,12,15,16,17]. Studies utilizing conventional SPECT/CT with sodium iodide crystal detectors noted substantially higher uptake values for normal spines, with mean SUVmax values ranging from 7.0 to 9.2 for mean SUVmax and mean SUVmean values from 4.6 to 7.3 [5,16,17]. In contrast, studies employing SPECT/CT with CZT detectors have reported relatively lower uptake values, showing mean SUVmax between 4.3 and 5.7 [12,15]. The present study found even lower uptake values for thoracic and lumbar spines, with mean SUVmax at 3.26 and 3.06, respectively, and mean SUVmean at 2.68 and 2.44. The previous studies conducted bone SPECT/CT with different radiopharmaceuticals, including ^99m^Tc-MDP [15,16,17], ^99m^Tc-3,3-diphosphono-1,2-propanodicarboxylic acid [5], and ^99m^Tc-hydroxydiphosphonate [12], each incorporating varying uptake times from 3 to 6 h and different SPECT/CT systems [5,12,15,16,17]. Therefore, it would be inappropriate to directly compare the uptake values of normal spines between the previous studies and our study. However, given the wide ranges of normal uptake values for spines across studies, using an uptake ratio with a reference normal bone site, instead of SUVmax, might be more appropriate for estimating bone lesion uptake in quantitative bone scintigraphy research involving various SPECT/CT scanners.

In addition to cardiovascular and cerebrovascular diseases, where SPECT/CT is commonly used, recent studies have highlighted its new potential applications in diverse clinical fields using a CZT camera [23,24,25]. In imaging for infectious diseases, SPECT/CT imaging with labelled leukocytes has proven clinically valuable for identifying infection sites and providing prognostic information [25,26]. For urologic diseases, dynamic three-dimensional diuretic renal scintigraphy with CZT SPECT/CT has shown high sensitivity and specificity in detecting ureteral obstruction [27]. The results of our study also confirmed the high diagnostic capability of bone scintigraphy in identifying bone metastasis of prostate cancer using a full-ring 360° CZT SPECT/CT system. Currently, prostate-specific membrane antigen PET/CT offers superior diagnostic performance for detecting metastasis in all stages of prostate cancer compared to conventional imaging modalities, thereby enhancing staging accuracy [28,29]. Additionally, recent studies have demonstrated the potential clinical role of fibroblast activation protein inhibitor PET/CT in detecting prostate cancer lesions with low prostate-specific membrane antigen expression [30]. Since bone SPECT/CT imaging has lower spatial resolution and sensitivity compared to PET/CT and cannot detect metastatic lesions in non-skeletal organs, SPECT/CT imaging’s future role may be limited [31]. However, considering the relatively low cost of SPECT/CT imaging and the broad availability of ^99m^Tc-labelled diphosphonate compounds, bone SPECT/CT is likely to maintain its clinical application in screening for bone metastasis in patients with prostate cancer. Furthermore, advances in SPECT/CT technology, including quantitative SPECT/CT analysis as demonstrated in this study, could help sustain its clinical impact [31].

The current study has several limitations. First, our research was conducted retrospectively at a single medical center, necessitating multi-center studies to externally validate the results. Second, only a relatively small number of patients (n = 82) were enrolled in this study, which might not sufficiently represent the variability in clinical presentations and imaging characteristics of prostate cancer patients with bone metastases. Third, the SUVmax and SUVmean measurements in this study were based on the patients’ body weight. Previous studies have shown that SUVs normalized by body surface area and skeletal volume are more effective in assessing bone lesion uptake compared to SUV based on body weight [16,32]. Therefore, future research is warranted to explore which SUV normalization methods can enhance the accuracy of quantitative SPECT. Finally, all bone lesions analyzed in this study were clinically diagnosed through imaging studies, and none were confirmed histopathologically. While obtaining histopathological confirmation for every bone lesion is impractical in clinical practice [33], the lack of such confirmation could lead to classification errors between metastatic and benign bone lesions, potentially affecting the results of the current study.

## 5. Conclusions

In this study, quantitative imaging parameters measured with a full-ring 360° CZT SPECT/CT system were significantly valuable in detecting bone metastases in patients with prostate cancer. Comparisons of the diagnostic performances between SUVmax and the lesion-to-normal bone uptake ratio parameters indicated the highest AUC value of 0.955 for the lesion-to-femur mean uptake ratio in differentiating bone metastases from benign bone lesions and normal bone uptake. By employing an optimal value of 5.38, the lesion-to-femur mean uptake ratio attained a sensitivity of 94.8% and a specificity of 86.5%. Quantitative SPECT/CT parameters could thus facilitate the characterization of metastatic bone lesions, thereby enhancing the diagnostic capabilities of bone SPECT/CT. Further studies are necessary to confirm the findings of this study.

## Figures and Tables

**Table 1:** Clinical characteristics of the patients.

Characteristics	Total Patients (*n* = 82)	Patients with Bone Metastasis (*n* = 31)	Patients with No Bone Metastasis (*n* = 51)	*p*-Value
Age (years) *	75 (49–87)	75 (49–86)	73 (57–87)	0.198
Serum PSA (ng/mL) *	36.7 (2.3–5000.0)	148.0 (2.8–5000.0)	18.8 (2.3–246.0)	<0.001
Gleason grade group				0.005
Group 1	3 (3.7%)	0 (0.0%)	3 (5.9%)	
Group 2	10 (12.2%)	1 (3.2%)	9 (17.6%)	
Group 3	8 (9.8%)	0 (0.0%)	8 (15.7%)	
Group 4	27 (32.9%)	11 (35.5%)	16 (31.4%)	
Group 5	34 (41.5%)	19 (61.3%)	15 (29.4%)	
T stage				<0.001
T2	28 (34.1%)	2 (6.5%)	26 (51.0%)	
T3	34 (41.5%)	16 (51.6%)	18 (35.3%)	
T4	20 (24.4%)	13 (41.9%)	7 (13.7%)	
N stage				<0.001
N0	58 (70.7%)	14 (45.2%)	44 (86.3%)	
N1	24 (29.3%)	17 (54.8%)	7 (13.7%)	
M stage				<0.001
M0	47 (57.3%)	0 (0.0%)	47 (92.2%)	
M1	35 (42.7%)	31 (100.0%)	4 (7.8%)	
TNM stage				<0.001
Stage II	16 (19.5%)	0 (0.0%)	16 (31.4%)	
Stage III	26 (31.7%)	0 (0.0%)	26 (51.0%)	
Stage IV	40 (48.8%)	31 (100.0%)	9 (17.6%)	

* Expressed as median (range). PSA, prostate-specific antigen.

**Table 2:** SUVmax and SUVmean of normal bone sites in the enrolled patients.

Normal Bone Sites	Total Patients (*n* = 82)	Patients with Bone Metastasis (*n* = 31)	Patients with No Bone Metastasis (*n* = 51)	*p*-Value
SUVmax				
Skull	1.29 (0.56–2.60)	1.09 (0.65–2.60)	1.32 (0.56–2.39)	0.464
Humerus	1.99 (0.85–4.00)	2.11 (0.85–4.00)	1.90 (0.99–3.25)	0.403
Thoracic spine	3.26 (1.35–5.99) ^1^	3.58 (1.95–5.70) ^4^	3.10 (1.35–5.99)	0.038
Lumbar spine	3.06 (1.32–6.12) ^2^	3.41 (1.32–6.12) ^5^	2.76 (1.78–5.57)	0.006
Iliac bone	2.78 (1.16–5.60) ^3^	3.38 (1.43–5.60) ^6^	2.65 (1.16–5.07)	0.022
Femur	1.88 (1.02–3.59)	1.85 (1.02–3.16)	1.91 (1.03–3.59)	0.928
SUVmean				
Skull	1.16 (0.52–2.43)	1.08 (0.54–2.43)	1.21 (0.52–2.09)	0.488
Humerus	1.30 (0.58–2.92)	1.40 (0.61–2.92)	1.29 (0.58–2.37)	0.556
Thoracic spine	2.68 (1.03–5.37) ^1^	3.00 (1.57–4.61) ^4^	2.56 (1.03–5.37)	0.058
Lumbar spine	2.44 (1.20–4.98) ^2^	2.68 (1.20–4.98) ^5^	2.26 (1.33–4.39)	0.018
Iliac bone	2.18 (0.86–4.50) ^3^	2.43 (0.92–4.23) ^6^	2.08 (0.86–4.50)	0.063
Femur	1.29 (0.65–2.29)	1.11 (0.65–2.29)	1.38 (0.68–2.14)	0.657

All data are expressed as median (range). ^1^ Measured in 76 patients; ^2^ Measured in 77 patients; ^3^ Measured in 78 patients; ^4^ Measured in 25 patients; ^5^ Measured in 26 patients; ^6^ Measured in 27 patients. SUVmax, maximum standardized uptake value; SUVmean, mean standardized uptake value.

**Table 3:** Comparison of SPECT/CT imaging parameters among metastatic bone lesions (n = 482), degenerative changes (n = 82), post-traumatic lesions (n = 48), and normal bone sites (n = 477).

Parameters	Metastatic Bone Lesions	Degenerative Changes	Post-Traumatic Lesions	Normal Bone Sites	*p*-Value
SUVmax	13.05 (9.48–17.67)	7.40 (6.55–8.90)	7.48 (5.48–15.22)	2.33 (1.66–3.10)	<0.001
Lesion-to-skull maximum uptake ratio	11.67 (8.13–18.89)	6.20 (4.51–8.54)	6.47 (4.19–10.73)	1.71 (1.18–2.42)	<0.001
Lesion-to-skull mean uptake ratio	13.23 (9.35–20.90)	7.32 (5.04–9.99)	7.46 (4.89–11.89)	1.93 (1.32–2.74)	<0.001
Lesion-to-humerus maximum uptake ratio	6.62 (4.76–11.03)	3.86 (2.90–5.43)	3.84 (2.16–6.21)	1.02 (0.91–1.48)	<0.001
Lesion-to-humerus mean uptake ratio	10.15 (6.23–15.12)	5.56 (4.52–8.40)	5.68 (3.15–9.69)	1.67 (1.32–2.21)	<0.001
Lesion-to-femur maximum uptake ratio	7.95 (5.21–11.59)	4.06 (3.19–5.35)	4.19 (2.57–6.94)	1.04 (0.94–1.53)	<0.001
Lesion-to-femur mean uptake ratio	12.29 (8.57–18.12)	6.10 (4.68–8.13)	6.31 (3.76–10.97)	1.65 (1.34–2.38)	<0.001

Data are presented as median (interquartile range). *p*-values result from the Kruskal–Wallis test. SUVmax, maximum standardized uptake value.

**Table 4:** Assessment of the diagnostic capability of SPECT/CT imaging parameters in detecting bone metastasis among metastatic (n = 482) and benign (n = 132) bone lesions and normal bone sites (n = 477).

Parameters	AUC (95% CI)	Cut-Off Value	Sensitivity (95% CI) (%)	Specificity (95% CI) (%)
SUVmax	0.949 (0.935–0.962)	5.11	98.3 (96.8–99.3)	79.5 (76.0–82.6)
Lesion-to-skull maximum uptake ratio	0.943 (0.928–0.956)	4.96	92.1 (89.3–94.4)	83.9 (80.7–86.7)
Lesion-to-skull mean uptake ratio	0.945 (0.930–0.958)	5.92	91.9 (89.1–94.2)	85.4 (82.3–88.1)
Lesion-to-humerus maximum uptake ratio	0.943 (0.927–0.956)	3.46	91.7 (88.9–94.0)	85.9 (82.9–88.5)
Lesion-to-humerus mean uptake ratio	0.873 (0.852–0.892)	5.09	82.8 (79.1–86.0)	85.7 (82.7–88.4)
Lesion-to-femur maximum uptake ratio	0.950 (0.935–0.962)	3.51	92.7 (90.0–94.9)	85.1 (82.0–87.8)
Lesion-to-femur mean uptake ratio	0.955 (0.942–0.967)	5.38	94.8 (92.4–96.6)	86.5 (83.6–89.1)

AUC, area under the receiver operating characteristic curve; CI, confidence interval; SUVmax, maximum standardized uptake value.

**Table 5:** Assessment of the diagnostic capability of SPECT/CT imaging parameters in distinguishing between metastatic (n = 482) and benign (n = 132) bone lesions.

Parameters	AUC (95% CI)	Cut-Off Value	Sensitivity (%)	Specificity (%)
SUVmax	0.775 (0.740–0.807)	8.96	79.5 (75.6–83.0)	71.2 (62.7–78.8)
Lesion-to-skull maximum uptake ratio	0.774 (0.739–0.806)	9.46	64.5 (60.1–68.8)	79.5 (71.7–86.6)
Lesion-to-skull mean uptake ratio	0.781 (0.746–0.813)	10.58	67.0 (62.6–71.2)	78.0 (70.0–84.8)
Lesion-to-humerus maximum uptake ratio	0.767 (0.731–0.800)	4.77	74.9 (70.8–78.7)	65.2 (56.4–73.2)
Lesion-to-humerus mean uptake ratio	0.699 (0.661–0.736)	9.69	53.1 (48.5–57.6)	81.8 (74.2–88.0)
Lesion-to-femur maximum uptake ratio	0.790 (0.755–0.821)	4.46	83.2 (79.6–86.4)	61.4 (52.5–69.7)
Lesion-to-femur mean uptake ratio	0.813 (0.780–0.843)	7.89	80.1 (76.2–83.6)	67.4 (58.7–75.3)

AUC, area under the receiver operating characteristic curve; CI, confidence interval; SUVmax, maximum standardized uptake value.

## Data Availability

The datasets presented in this study are available on request from the corresponding author due to ethical restrictions.
